# Inter-observer Variability in the Analysis of CO-RADS Classification for COVID-19 Patients

**DOI:** 10.3390/tropicalmed8120523

**Published:** 2023-12-17

**Authors:** Yassir Edrees Almalki, Mohammad Abd Alkhalik Basha, Maha Ibrahim Metwally, Ahmed Mohamed Housseini, Sharifa Khalid Alduraibi, Ziyad A. Almushayti, Asim S. Aldhilan, Mahmoud Mohamed Elzoghbi, Esraa Attia Gabr, Esaraa Manajrah, Reham Mohammed Farid Hijazy, Loujain Mohamed Khear Akbazli, Ayman El Mokadem, Ahmed M. A. Basha, Walid Mosallam

**Affiliations:** 1 Division of Radiology, Department of Internal Medicine, Medical College, Najran University, Najran 61441, Saudi Arabia; 2 Department of Diagnostic Radiology, Faculty of Human Medicine, Zagazig University, Zagazig 44519, Egypt; maatya@zu.edu.eg (M.A.A.B.); mimetwally@zu.edu.eg (M.I.M.); 3 Department of Radio-Diagnosis, Faculty of Human Medicine, Suez Canal University, Esmaelia 41522, Egypt; housseini2008@gmail.com (A.M.H.); mahmoudelzoghby@med.suez.edu.eg (M.M.E.); pgs.000933397@med.suez.edu.eg (E.A.G.); walidmosallam@med.suez.edu.eg (W.M.); 4 Department of Radiology, College of Medicine, Qassim University, Buraidah 52571, Saudi Arabia; salduraibi@qu.edu.sa (S.K.A.); ziyadalmushayti@qu.edu.sa (Z.A.A.); a.aldhilan@qu.edu.sa (A.S.A.); 5 Faculty of Human Medicine, Suez Canal University, Esmaelia 41522, Egypt; esraa.manajrah@hotmail.com (E.M.); reham_h@live.com (R.M.F.H.); loujainmohammad@hotmail.com (L.M.K.A.); 6 Department of Pulmonary Medicine, Faculty of Human Medicine, Suez Canal University, Esmaelia 41522, Egypt; ayman.elmokadem@med.suez.edu.eg; 7 Faculty of General Medicine, Saint Petersburg State University, Egypt Branch, Cairo 11646, Egypt; ahm7edbasha@gmail.com

**Keywords:** COVID-19, CO-RADS, CT chest, inter-observer, variability

## Abstract

During the early stages of the pandemic, computed tomography (CT) of the chest, along with serological and clinical data, was frequently utilized in diagnosing COVID-19, particularly in regions facing challenges such as shortages of PCR kits. In these circumstances, CT scans played a crucial role in diagnosing COVID-19 and guiding patient management. The COVID-19 Reporting and Data System (CO-RADS) was established as a standardized reporting system for cases of COVID-19 pneumonia. Its implementation necessitates a high level of agreement among observers to prevent any potential confusion. This study aimed to assess the inter-observer agreement between physicians from different specialties with variable levels of experience in their CO-RADS scoring of CT chests for confirmed COVID-19 patients, and to assess the feasibility of applying this reporting system to those having little experience with it. All chest CT images of patients with positive RT-PCR tests for COVID-19 were retrospectively reviewed by seven observers. The observers were divided into three groups according to their type of specialty (three radiologists, three house officers, and one pulmonologist). The observers assessed each image and categorized the patients into five CO-RADS groups. A total of 630 participants were included in this study. The inter-observer agreement was almost perfect among the radiologists, substantial among a pulmonologist and the house officers, and moderate-to-substantial among the radiologists, the pulmonologist, and the house officers. There was substantial to almost perfect inter-observer agreement when reporting using the CO-RADS among observers with different experience levels. Although the inter-observer variability among the radiologists was high, it decreased compared to the pulmonologist and house officers. Radiologists, house officers, and pulmonologists applying the CO-RADS can accurately and promptly identify typical CT imaging features of lung involvement in COVID-19.

## 1. Introduction

The novel coronavirus (SARS-CoV-2) originating from Wuhan, China, has emerged as a significant global threat [1,2,3,4]. The reverse transcription-polymerase chain reaction (RT-PCR) assay is the gold standard for diagnosing COVID-19, but its sensitivity can vary between 42% and 83%, depending on the viral load [5]. Furthermore, the limited availability of RT-PCR tests and delayed delivery of results in some developing countries posed challenges during the initial wave of the pandemic [5,6].

Chest computed tomography (CT) plays a crucial role in the diagnosis and management of COVID-19 pneumonia [7,8]. While COVID-19 exhibits specific CT features, it can overlap with other diseases, particularly viral pneumonia [9]. Consequently, there is a degree of variability among radiologists when interpreting and classifying COVID-19 imaging findings [10,11]. Initial studies assessing chest CT findings in COVID-19 patients reported high sensitivity (94%) but low specificity (37%) [12,13,14]. To address these challenges and ensure consistent reporting, the Dutch Radiological Society introduced the COVID-19 Reporting and Data System (CO-RADS) in early March 2020. The CO-RADS provides a categorical assessment scale ranging from CO-RADS 1 (negative for pneumonia) to CO-RADS 5 (indicative of typical COVID-19) to standardize radiologists’ interpretations [9,15]. However, recent studies have highlighted significant inter-observer variability in CO-RADS categorization [15,16].

Understanding the extent of inter-observer variability in the CO-RADS classification is crucial for ensuring consistent and reliable application of this reporting system. Therefore, in this study, we aimed to assess the inter-observer agreement among physicians from different specialties with varying levels of experience in their CO-RADS scoring of CT chests from confirmed COVID-19 patients. Furthermore, we evaluated the feasibility of implementing this reporting system among physicians with limited knowledge.

## 2. Materials and Methods

### 2.1. Study Participants

All chest CT images of patients with positive RT-PCR tests for COVID-19 from the medical records of Suez Canal University Hospitals from August 2020 to June 2021 were eligible for this study. Exclusion criteria included patients with at least one negative RT-PCR test (*n* = 5), patients with unavailable RT-PCR results (*n* = 14), and CT scans with major artefacts such as respiratory motion or incomplete scanning that affected the accuracy of the CT image interpretation (*n* = 8). After applying the inclusion and exclusion criteria, 630 patients were included in this study.

The study was approved by the Institutional Review Board, and consent was waived due to the retrospective nature of the study.

### 2.2. CT Chest Imaging

Patients underwent CT without contrast using the same protocol with a single 64-slice CT scanner (LightSpeed VCT; GE Healthcare; Chicago; United States). The acquisition parameters at our hospital were as follows: 120 kV tube voltage with automatic tube current modulation (150 mAs); tube rotation time of 0.28 s; beam collimation of 128 ch × 0.6 mm; and beam pitch of 1.5. By default, 2.0 mm without interslice gap chest CT images were reconstructed using a sharp tissue kernel (Bl57) with the filtered back-projection technique. The slice thickness of the reconstructed images ranged from 1.25 to 5 mm at other institutions.

### 2.3. Imaging Analysis

Each chest CT image was reviewed by seven observers who were divided into three groups according to the type of specialty, as follows: The radiologist group included observer 1 (a chest consultant radiologist with 20 years of experience), observer 2 (a radiologist consultant with ten years of experience), and observer 3 (a radiologist with five years of experience). The chest physician group included observer 4 (a chest consultant with 15 years of experience). The foundation-year physician group included observers with five, six, and seven foundation-year physicians with little experience. The foundation-year physician group was the only group that received a training session by the main researcher, which included one hour of conceptual lectures followed by practical application on 30 CTs performed on COVID-19 patients with findings corresponding to each CO-RADS category. These thirty cases of training were excluded from the sample study. For each patient, the chest CT scans were evaluated for the following characteristics: presence, amount, and distribution pattern of ground-glass opacities; the presence of consolidation; the presence of air bronchograms; the number of lobes affected where ground-glass or consolidative opacities were present; the presence of nodules; the presence of pleural effusion; the presence of thoracic lymphadenopathy (defined as lymph node size > 10 mm in short axis size), airway abnormalities (including airway wall thickening, bronchiectasis, and endoluminal secretions); and the presence of underlying lung diseases such as emphysema or fibrosis. Opacities with a crazy-paving pattern, a reverse halo sign, rounded morphology, intralesional cavitation, and linear opacities were noted. The observers assessed each image and categorized the patients according to the CO-RADS classification system (Table 1) [15]. 

The extracted chest CT images were anonymized and the observers were blinded to all clinical data of the patients, including the PCR results, except for their age and sex. The radiologist plotted the detailed descriptive data for each patient and identified the CO-RADS classes in the tables. However, the different groups plotted the cases according to the CO-RADS classification without detailed descriptive data.

### 2.4. Statistical Analysis

Statistical analyses were performed using SPSS version 26 (IBM, Armonk, NY, USA). The data are presented in tables and figures. Qualitative data are presented as frequencies and percentages. To determine the inter-observer agreement, the Fleiss k value was determined across the observers. The k values were obtained by comparing the CO-RADS scores of each observer with the median scores of the remaining seven observers. Inter-observer agreement was considered slight for a k value of 0.01–0.20, fair for a k value of 0.21–0.40, moderate for a k value of 0.41–0.60, substantial for a k value of 0.61–0.80, and almost perfect for a k value of 0.81–1.00 [17]. A probability value of less than 0.05 was considered statistically significant for all tests.

## 3. Results

A total of 630 participants were included in this study. Table 2 provides an overview of the basic characteristics of the study participants, including their gender and nationality. Among the participants, the majority were male, accounting for 61.9% (390 patients), while the remaining 38.1% were female (240 patients). Regarding nationality, the study primarily included Egyptian participants, who accounted for 98.7% (622) of the patients. There were also a small number of participants from other nationalities, including three patients (0.5%) from Italy, one patient (0.2%) from India, two patients (0.3%) from Germany, one patient (0.2%) from the United States, and one patient (0.2%) from Ukraine.

All of the chest CT scans were assessed by the three radiologists, and Table 3 provides an overview of their findings. The CT scans were assessed as normal for 107, 104, and 103 patients by observers 1, 2, and 3, respectively. Emphysema was found in 16, 13, and 13 patients by observers 1, 2, and 3, respectively. In addition, lung masses were found in 12, 6, and 6 patients by observers 1, 2, and 3, respectively. This table provides additional details on the number of CT scans showing peri-fissural nodules, tree-in-bud, centrilobular nodules, consolidation, cavitation, and smooth septal thickening with pleural effusion, as identified by each observer.

Table 4 focuses on ground glass opacity (GGO) characteristics observed by the radiologists. The GGOs were perihilar in 349, 334, and 319; single foci in 20, 403, and 13; centrilobular in 405, 14, and 371; and homogenous extensive in 18, 109, and 11 by observers 1, 2, and 3, respectively. GGOs with smooth septal thickening were found in 113, 2, and 114 patients, and smooth septal thickening and effusion were found in 5, 149, and 2 by observers 1, 2, and 3, respectively. Small GGOs, not centrilobular, and not close to the pleura were found in 152, 4, and 143 by observers 1, 2, and 3, respectively. Organizing (scaring) pneumonia patterns without typical features were found in 9, 366, and 4 by observers 1, 2, and 3, respectively. Multifocal bilateral GGOs were found in 375, 396, and 370 by observers 1, 2, and 3, respectively. Multifocal unilateral GGOs close to the pleural surface or fissure were found in 404, 404, and 409 by observers 1, 2, and 3, respectively. GGOs with typical features on one side and unifocal on the other were found in 15, 15, and 14 by observers 1, 2, and 3, respectively. Unifocal bilateral GGOs were found in 6, 6, and 3 by observers 1, 2, and 3, respectively.

Table 5 highlights the typical features identified by the radiologists, including multifocal bilateral GGOs with consolidation, organizing (scaring) pneumonia, crazy-paving signs, thickened vessels, and reversed halo signs. Multifocal bilateral GGOs with consolidation close to the pleural surface or fissure and pleural sparing were found in 385, 382, and 369 by observers 1, 2, and 3, respectively. The typical features of organizing (scaring) pneumonia patterns were found in 291, 283, and 292 by observers 1, 2, and 3, respectively. Typical features with crazy paving were found in 108, 97, and 109 by observers 1, 2, and 3, respectively. Typical features with thickened vessels were found in 355, 308, and 362 by observers 1, 2, and 3, respectively. Typical features with reversed halos were found in 39, 35, and 34 by observers 1, 2, and 3, respectively.

Table 6 shows the results of applying the CO-RADS classification system to the CT scans by the three radiologists. Observer 1 classified 21.1% as CO-RADS 1, 2.4% as CO-RADS 2, 5.7% as CO-RADS 3, and 10.5% as CO-RADS 4. Observer 2 recorded very similar results, with 21.1% CO-RADS 1, 2.4% CO-RADS 2, 5.7% CO-RADS 3, and 10.6% CO-RADS 4. Observer 3 classified 21.6% as CO-RADS 1, 2.2% as CO-RADS 2, 5.1% as CO-RADS 3, and 8.3% as CO-RADS 4. All three observers classified the majority of the cases as CO-RADS 5, with percentages ranging from 60.2% to 62.9%. The level of agreement between these observers was very high, based on the Fleiss kappa values. The Fleiss kappa value was 0.997 between observers 1 and 2, 0.921 between observers 2 and 3, and 0.924 between observers 1 and 3. This indicates an almost perfect consensus between the observers when applying the CO-RADS classification to the scans.

Table 7 extends the CO-RADS analysis to include a pulmonologist (observer 4) and house officers (observers 5, 6, and 7). The reported percentages of CO-RADS classifications by these observers were as follows: Observer 4 reported 21.7% CO-RADS 1, 8% CO-RADS 2, 8.6% CO-RADS 3, 11.3% CO-RADS 4, and 57.6% CO-RADS 5. Observer 5 reported 21.3% for CO-RADS 1, 0.6% for CO-RADS 2, 3.2% for CO-RADS 3, 6.2% for CO-RADS 4, and 68.8% for CO-RADS 5. The extent of agreement among the observers was evaluated using the Fleiss kappa value. The inter-observer variability was substantial among the observers, with κ values ranging from 0.636 to 0.736. However, the agreement between observers 6 and 7 was rated as moderate, with a κ value of 0.584.

Table 8 provides an overview of the inter-observer agreement among radiologists, the pulmonologist, and house officers regarding the CO-RADS classification. Among the radiologists and the pulmonologist, there was a substantial inter-observer agreement, indicated by κ values ranging from 0.613 to 0.661. In the case of the radiologists and house officers, the inter-observer agreement was moderate to substantial, with κ values ranging between 0.503 and 0.692. Notably, the agreement between radiologists and observer 6 was almost perfect, with κ values ranging from 0.900 to 0.903. Overall, the table demonstrates moderate to substantial inter-observer agreement on CO-RADS classifications among the different medical professionals.

**Figure 1:**
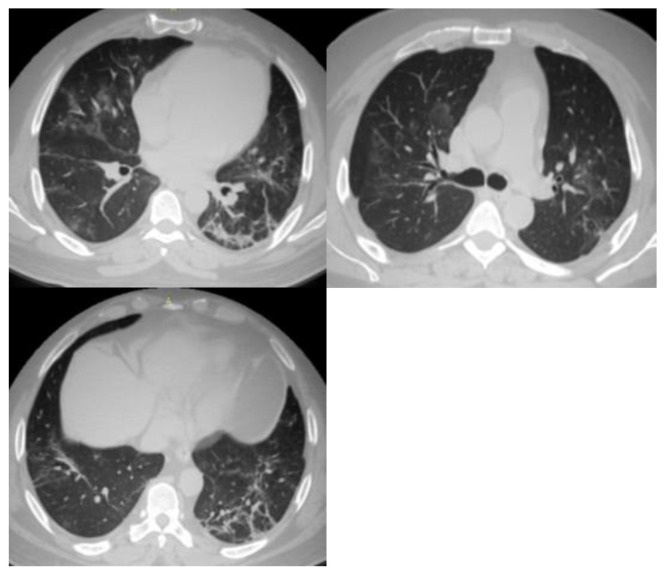
Multiple chest CT images of COVID-19 patients. All observers agreed to calculate the CO-RADS for this case as CO-RADS 5. The agreement between radiologists and pulmonologist was based on the following features: Typical features of multifocal bilateral GGO and consolidation, peri-fissural nodules, perihilar GGO, and centrilobular GGO. In addition to the previous findings, all radiologists agreed on the typical features of multifocal bilateral GGO, consolidation, organizing pneumonia, and thickened vessels.

**Figure 2:**
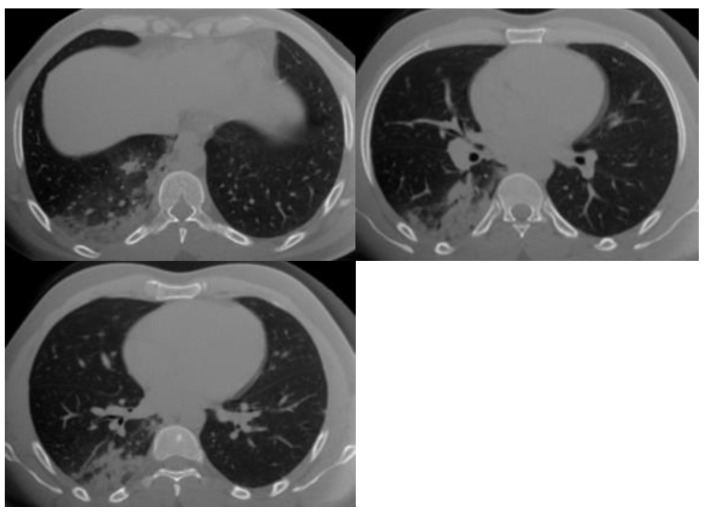
Multiple chest CT images of COVID-19 patients. Two senior radiologists, a pulmonologist, and a house officer agreed to calculate the CO-RADS for this case as CO-RADS 4. Agreement was based on the following features: multifocal unilateral GGO and consolidation close to the pleura. One junior radiologist with another house officer agreed to calculate the CO-RADS for this case as CO-RADS 5. The agreement was based on the following features: multifocal unilateral GGO, consolidation close to the pleura, and other side unifocal GGO.

**Figure 3:**
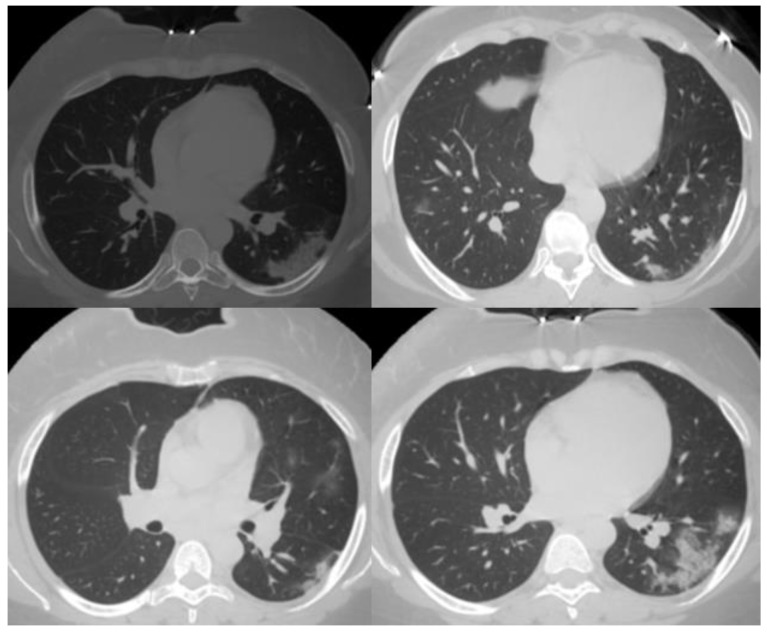
Multiple chest CT images of COVID-19 patients. Senior radiologists and the pulmonologist agreed to calculate the CO-RADS for this case as CO-RADS 4. The agreement was based on the following features: typical features of multifocal unilateral GGO, consolidation, unifocal GGO on the other side, organizing pneumonia without typical features, multiple unilateral ventrilobular GGO, and focal unilateral GGO. The junior radiologist and house officers agreed to calculate the CO-RADS for this patient as CO-RADS 5. The agreement was based on the following features: multifocal bilateral GGO and consolidation with organizing pneumonia.

Representative cases from this study are shown in Figure 1, Figure 2 and Figure 3.

## 4. Discussion

CT imaging is widely used as a diagnostic method for COVID-19 pneumonia. Radiological differential diagnosis and isolation of other viral agents causing pneumonia in patients have gained importance, especially during pandemics [18]. In many countries, CT scans, together with serological and clinical data, are commonly used to diagnose COVID-19. Therefore, a CT imaging protocol is required to enhance radiation protection and achieve the ALARA radiation rule [19]. Although chest CT findings may partially overlap with other diseases, particularly other types of viral infections, COVID-19 may have specific CT characteristics that are less common under different conditions [20].

The current study assessed the inter-observer agreement in applying the CO-RADS classification to interpret the chest CT scans of 630 patients. Our results demonstrated substantial to almost perfect agreement between radiologists, a pulmonologist, and house officers in classifying COVID-19 severity using the CO-RADS. Specifically, there was an almost perfect agreement between the three radiologists (κ = 0.921–0.997). This indicates that the CO-RADS allows radiologists to consistently classify COVID-19 severity on CT scans. Agreement was slightly lower but still substantial between the pulmonologist and the three house officers (κ = 0.584–0.736). This suggests that pulmonologists and house officers can also reliably apply the CO-RADS, although there is more variability compared to specialized radiologists. These results are consistent with those of previous studies [3,5,13,15,16,21,22,23,24,25,26]. Fonseca et al. [3] emphasized the substantial inter-observer agreement among the three readers for CO-RADS classifications, even three months after the initial case analysis, and without any additional training (κ = 0.642). Özdemir et al. [5] reported good to almost perfect inter-observer variability among their four readers (κ = 0.79–0.86). Prokop et al. [15] reported an overall moderate reliability among their eight readers (κ = 0.47). Bellini et al. [16] registered an overall moderate inter-observer agreement for CO-RADS ratings among 12 readers (κ = 0.43). Fujioka et al. [21] reported substantial to almost perfect levels of inter-observer agreement for the CO-RADS (ICC = 0.800–0.874). Sheha et al. [22] discovered that CO-RADS reporting exhibited good inter-rater agreement (ICC = 0.75). Abdel-Tawab et al. [23] reported an overall excellent inter-reviewer agreement among their three readers for the CO-RADS (κ = 0.801). Nair et al. [24] reported an overall moderate inter-observer agreement for CO-RADS categories among the six readers (κ = 0.548). Atta et al. [25] reported an overall substantial agreement among three readers (κ = 0.78). Sushentsev et al. [26] demonstrated moderate inter-observer agreement among the three readers for the CO-RADS, with a κ value of 0.51.

In the present study, assessing the agreement between radiologists, a pulmonologist, and house officers indicated moderate to substantial agreement (κ = 0.503–0.692). The highest agreement was observed between the radiologists and one house officer (observer 6). Nonetheless, it is worth noting that, overall, there was a reasonable level of agreement among reviewers with varying levels of experience. The radiologists demonstrated perfect inter-observer agreement, while the less experienced house officers showed moderate agreement with the radiologists. These findings suggest that while experience may contribute to higher agreement levels, clinicians with varying levels of experience can still provide meaningful assessments of COVID-19 CT images. This aligns with the findings of Fonseca et al. [3].

Although the present study demonstrated good inter-observer agreement among radiologists, a pulmonologist, and house officers, it is crucial to address the factors contributing to inter-observer variability. Inter-observer variability in the CO-RADS may be attributed to several factors. Firstly, the imaging features of COVID-19 have a wide spectrum, and may overlap with other diagnoses [27]. Secondly, CO-RADS descriptors are subjective and qualitative [13]. Thirdly, many radiologists were initially unfamiliar with CO-RADS [9]. Finally, inherent differences exist among radiologists in diagnostic reasoning and image interpretation [28]. Standardized training in the CO-RADS, calibration exercises, and prudent use of its ordinal scale may help improve agreement and consistency. Nonetheless, inter-observer variability underscores the complexity and challenges of classifying COVID-19 pneumonia [27]. 

Similar to our findings, many studies agree that the most frequently observed characteristic results of COVID-19 are multifocal bilateral, peripherally located ground-glass appearance, and peripheral consolidation close to the pleural surface or fissure with pleural sparing [5,11,18,29].

A notable finding in our study was the high disagreement between radiologists in fundamental radiological findings. These discrepancies may be attributed to several factors, including varying definitions and interpretation criteria, subjective interpretations, varying experience levels among radiologists, and radiologists potentially being influenced by fatigue when reading large numbers of chest CT scans. Additional factors that may contribute to disagreements include the quality of the scan itself and the possibility of overlooking subtle abnormalities. Going forward, steps could be taken to standardize the evaluation criteria, provide more training opportunities to improve consistency, implement quality checks to catch discrepancies, and ensure radiologists take breaks during long review sessions.

Regarding the CO-RADS, higher proportions of COVID-19 patients were CO-RAD 5 and 1. Sheha et al. [22] reported similar results for RT-PCR-confirmed cases, with CO-RADS categories 1 and 5 showing a higher proportion of positive cases than the CO-RADS 2 category. This also agrees with the results reported by De Jaegere et al. [30]. 

In summary, the results of this study provide insights into the characteristics observed in chest CT scans, the distribution of CO-RADS categories, and the inter-observer agreement among radiologists, pulmonologists, and house officers. The high inter-observer agreement supports the use of the CO-RADS classification for systematically assessing and communicating the spectrum of COVID-19 findings on CT scans. The system allows for consistent interpretation between radiologists, as well as substantial agreement between specialties. Moreover, continuous refinement and validation of the CO-RADS methodology and descriptors are essential to improve its accuracy and reliability. As new knowledge and evidence emerge regarding the imaging features of COVID-19, updates to the classification system can be made to ensure its relevance and effectiveness.

The current study had certain limitations. Firstly, it focused exclusively on patients with PCR-confirmed COVID-19, and lacked a control group with alternative respiratory diagnoses. Without such a group, we were unable to fully determine the accuracy of the CO-RADS in distinguishing COVID-19 from other respiratory conditions. Further research should evaluate the specificity and predictive values of CO-RADS scoring by including patients with confirmed negative COVID-19 test results. This will allow for a more rigorous assessment of the classification system’s ability to correctly diagnose COVID-19 and avoid false positive errors. Secondly, the study was constrained by its retrospective nature, and there was a lack of clinical data regarding the duration of symptoms at the time of CT scanning. Thirdly, the small number of observers in our study may potentially impact the generalizability and reproducibility of our findings. Therefore, future studies with a larger number of observers are required to validate and strengthen our findings. Fourthly, observer experience could be a potential confounding factor in interpreting our study results. However, we attempted to diminish this issue through rigorous training and standardization of data collection procedures for all observers. This included providing clear CO-RADS classification guidelines, establishing criteria for making scoring judgements, and promoting consistency in data collection techniques across radiologists of varying seniority levels. However, some residual variability due to subjective interpretations still likely remained. Further research quantifying the impact of radiologist credentials and experience on CO-RADS scoring reliability would provide additional clarity. Finally, the time interval between the initial PCR tests and CT scans was not strictly defined, which could contribute to discrepancies between PCR-based diagnoses and the observed imaging patterns, particularly at different stages of the disease course. Addressing this limitation in future research is crucial for obtaining a more comprehensive understanding of the relationship between CT imaging and COVID-19 diagnosis.

## 5. Conclusions

The present study revealed almost perfect inter-observer agreement when reporting the use of the CO-RADS among radiologists with varying levels of experience. Although the inter-observer variability of the CO-RADS classification system for COVID-19 among radiologists was high, it decreased compared to the pulmonologist and house officers. Radiologists, house officers, and pulmonologists can apply the CO-RADS accurately to promptly identify typical CT imaging features of lung involvement in COVID-19. 

## Figures and Tables

**Table 1:** CO-RADS categories.

CO-RADS	Level of Suspicion	CT Findings
1	Very low	Normal or non-infectious CT findings.
2	Low	CT findings incompatible with COVID-19: bronchitis, infectious bronchiolitis, and bronchopneumonia.
3	Equivocal/uncertain	CT findings of other viral pneumonia or non-infectious results: perihilar GGO, homogenous extensive GGO, and GGO with smooth interlobular septal thickening.
4	High	CT findings are similar to those for CO-RADS 5, but a lack of contact with the visceral pleura, located unilaterally, in a peri-broncho vascular distribution, or when the findings are superimposed on pre-existing lung abnormalities.
5	Very high	Typical CT findings: ground-glass opacities with or without consolidations in lung regions close to visceral pleural surfaces and multifocal bilateral distribution.

CO-RADS, COVID-19 Reporting and Data System; CT, computed tomography; COVID-19, coronavirus disease 2019; GGO, ground-glass opacities.

**Table 2:** Basic characteristics of the participants.

Variable		*n* (%)
**Gender**	Male	390 (61.9)
	Female	240 (38.1)
**Nationality**	Egypt	622 (98.7)
	Italian	3 (0.5)
	Indian	1 (0.2)
	German	2 (0.3)
	American	1 (0.2)
	Ukrainian	1 (0.2)

**Table 3:** Characteristics of chest CT performed by radiologists.

Variable	Observer 1	Observer 2	Observer 3
**Normal**	107	104	103
**Emphysema**	16	13	13
**Peri-fissural nodule**	56	52	52
**Lung mass**	12	6	6
**Tree in bud**	7	2	3
**Centrilobular nodule**	53	49	44
**Consolidation**	68	63	63
**Cavitation**	5	1	1
**Smooth septal thickening with pleural effusion**	8	3	4

**Table 4:** Ground glass opacity characteristics of radiologists.

Variable	Observer 1	Observer 2	Observer 3
**Perihilar**	349	334	319
**Single focus**	20	403	13
**Centrilobular**	405	14	371
**Homogenous extensive**	18	109	11
**With smooth septal thickening**	113	2	114
**With smooth septal thickening and effusion**	5	149	2
**Small, not centrilobular, and not close to pleura**	152	4	143
**Organizing (scaring) pneumonia pattern without typical features**	9	366	4
**Multifocal bilateral**	375	396	370
**Multifocal unilateral close to pleural surface or fissure**	404	404	409
**Typical features on one side and unifocal other side**	15	15	14
**Unifocal bilateral**	6	6	3

**Table 5:** Typical features and associated characteristics.

Variable	Observer 1	Observer 2	Observer 3
**Typical features: Multifocal bilateral GGOs, consolidation, close to the pleural surface or fissure, and pleural sparing**	385	382	369
**Typical features with organizing (scaring) pneumonia pattern**	291	283	292
**Typical features with crazy paving**	108	97	109
**Typical features with thickened vessels**	355	308	362
**Typical features with reversed halo**	39	35	34

**Table 6:** Radiologist CO-RADS.

Variable		Observer 1	Observer 2	Observer 3
**CO-RADS**	1	133 (21.1)	133 (21.1)	136 (21.6)
	2	15 (2.4)	15 (2.4)	14 (2.2)
	3	36 (5.7)	36 (5.7)	32 (5.1)
	4	66 (10.5)	67 (10.6)	52 (8.3)
	5	380 (60.3)	379 (60.2)	396 (62.9)
**κ**		0.997 ^a^ (0.866–1.00)	0.921 ^b^ (0.790–1.00)	0.924 ^c^ (0.789–1.00)
***p*-value**		<0.001	<0.001	<0.001

κ, Cohen’s kappa coefficient (95% confidence interval); ^a^, observers 1 and 2; ^b^, observers 2 and 3; ^c^, observers 1 and 3.

**Table 7:** CO-RADS among house officers and pulmonologist.

Variable		Observer 4	Observer 5	Observer 6	Observer 7	*p*-Value
**CO-RADS**	1	137 (21.7)	134 (21.3)	145 (23)	150 (23.8)	<0.001
	2	5 (8)	4 (0.6)	13 (2.1)	2 (0.3)	
	3	54 (8.6)	20 (3.2)	32 (5.1)	34 (5.4)	
	4	71 (11.3)	39 (6.2)	55 (8.7)	37 (5.9)	
	5	363 (57.6)	433 (68.8)	385 (61.1)	407 (64.6)	
**κ**	0.661 ^a^ (0.530–0.792)	0.636 ^b^ (0.505–0.767)	0.584 ^c^ (0.453–0.715)	0.676 ^d^ (0.545–0.807)	0.621 ^e^ (0.490–0.752)	0.736 ^f^ (0.605–0.867)

κ, Cohen’s kappa coefficient (95% confidence interval); ^a^, observer 4 and 5; ^b^, observer 5 and 6; ^c^, observer 6 and 7; ^d^, observer 4 and 6; ^e^, observer 4 and 7; ^f^, observer 5 and 7.

**Table 8:** Agreement variability between radiologists, pulmonologist, and house officers regarding the CO-RADS.

Variable	Observer 1	Observer 2	Observer 3
**Observer 4**	0.615 (0.484–0.746)	0.613 (0.482–0.744)	0.661 (0.530–0.792)
**Observer 5**	0.672 (0.541–0.803)	0.669 (0.538–0.800)	0.692 (0.561–0.823)
**Observer 6**	0.903 (0.772–1.00)	0.900 (0.769–1.00)	0.903 (0.772–1.00)
**Observer 7**	0.556 (0.425–0.687)	0.554 (0.423–0.685)	0.503 (0.371–0.634)

The values represent Cohen’s kappa coefficient (95% confidence interval).

## Data Availability

The CT and clinical datasets used and/or analyzed during the current study are available from the corresponding author upon reasonable request.
